# Predicting Post-Radiotherapy Lymphocyte Recovery for Individualized Risk Stratification in Locally Advanced Esophageal Squamous Cell Carcinoma

**DOI:** 10.3390/curroncol33060374

**Published:** 2026-06-22

**Authors:** Hongshan Ji, Yuhao Su, Menglu Liu, Yajing Wang, Qiuying An, Yage Jia, Zihan Zhang, Jin Yan, Jingxin Bai, Ping Zhang, Zhiguo Zhou

**Affiliations:** Department of Radiation Oncology, The Fourth Hospital of Hebei Medical University, Shijiazhuang 050011, China; hongshanji0731@126.com (H.J.); 22034101418@stu.hebmu.edu.cn (Y.S.); 25033100559@stu.hebmu.edu.cn (M.L.); wang_yj96@163.com (Y.W.); anqy1997@hebmu.edu.cn (Q.A.); 23033100478@stu.hebmu.edu.cn (Y.J.); 24034101434@stu.hebmu.edu.cn (Z.Z.); 24034101435@stu.hebmu.edu.cn (J.Y.); 17831188165@163.com (J.B.); zhangping0320@hebmu.edu.cn (P.Z.)

**Keywords:** lymphocyte recovery, esophageal squamous cell carcinoma, radiotherapy, radiation-induced lymphopenia, individualized risk stratification, prediction model

## Abstract

The prognostic value of post-radiotherapy (RT) lymphocyte recovery remains unclear in locally advanced esophageal squamous cell carcinoma (ESCC), and tools to predict recovery are lacking. This study evaluated lymphocyte recovery as a survival predictor and developed a prediction model. We analyzed 233 patients (2019–2024; training:validation = 7:3). Lymphocyte recovery was assessed at 1 and 3 months post-RT (ΔALC_1_ > 0.41 and ΔALC_3_ > 0.25 × 10^9^/L, calculated as ALC at each time point minus ALC at the end of RT). Patients were stratified into three groups by recovery status: no recovery (Group 0), recovery at both time points (Group 2), or at only one time point (Group 1). Multivariate logistic regression identified predictors of lymphocyte recovery, and a nomogram was developed and internally validated. Median overall survival (OS) was 26.4 months and median progression-free survival (PFS) was 13.9 months. OS differed significantly among groups: 16.0 months (Group 0), 26.0 months (Group 1), and 50.0 months (Group 2) (*p* < 0.001). Median PFS was 10.2, 12.0, and 36.6 months, respectively (*p* < 0.001). Independent predictors included ECOG 0 and thoracic spine V_5_ < 57.3%; planning target volume < 210 cm^3^ showed a trend toward association (*p* = 0.051). The nomogram demonstrated AUCs of 0.77 and 0.75 in the training and validation cohorts. Superior lymphocyte recovery appears to be associated with improved survival. The model, if externally validated, may facilitate individualized risk stratification.

## Simple Summary

Radiotherapy for locally advanced esophageal squamous cell carcinoma often depletes immune cells called lymphocytes, which are essential in anti-tumor immunity. But whether these cells recover after radiotherapy and how this affects patient survival remain unclear. This study of 233 patients found that better lymphocyte recovery at 1 and 3 months after radiotherapy was associated with significantly longer survival. We developed a model using patients’ performance status, radiotherapy planning target volume, and radiation dose to the thoracic spine to predict which patients are likely to recover well. This tool may help identify patients at high risk of poor lymphocyte recovery. If externally validated, it could inform hypotheses for radiation planning to spare lymphocyte-rich organs, though whether this improves outcomes requires prospective testing.

## 1. Introduction

The combination of radiotherapy (RT) or chemoradiotherapy (CRT) with immunotherapy has been incorporated into the treatment paradigm for locally advanced esophageal squamous cell carcinoma (ESCC) [1,2,3,4], highlighting the critical role of the host immune system in cancer control. However, RT inevitably depletes circulating lymphocytes, which are essential for anti-tumor immunity. Radiation-induced lymphopenia (RIL) has been shown to compromise treatment efficacy and correlate with poor survival outcomes in esophageal cancer [5,6,7,8,9]. Consequently, dose constraints to lymphocyte-rich organs at risk (OARs)—such as the thoracic spine and spleen—have drawn increasing attention [7,8,10,11,12].

Conversely, the prognostic impact of post-RT lymphocyte recovery remains poorly characterized. Existing studies have reported inconsistent findings regarding the optimal timing for assessment, the definition of recovery, and the dose-volume parameters of lymphocyte-rich organs at risk (OARs). Cheung et al. assessed lymphocyte recovery at 6 months post-RT using the absolute lymphocyte count (ALC) recovery index (ARI), defined as the ratio of ALC at 6 months post-RT to pre-RT ALC, with ARI ≥ 65% indicating satisfactory recovery [8]. They identified thoracic spine V_35_ as an independent predictor of ARI, with a cut-off of 25.7%, and demonstrated that high ARI was prognostic for improved overall survival (OS) and progression-free survival (PFS) [8]. In contrast, Deng et al. evaluated recovery at 6–8 weeks post-CRT using an absolute ALC threshold of ≥0.8 × 10^9^/L and found no significant association with OS or PFS in esophageal cancer patients with severe radiation-induced lymphopenia [13]. Tseng et al. similarly used the 6-month ARI (≥60%) and reported that inadequate lymphocyte recovery was significantly associated with worse OS and local recurrence-free survival; bone marrow V_5_ was identified as an independent predictor [14]. Notably, our previous study defined recovery using ΔALC (ALC at 3 months post-RT minus ALC at the end of RT (EoRT)) and demonstrated its prognostic value for OS and PFS in locally advanced ESCC [15]. These discrepancies in assessment timing—from 6–8 weeks to 6 months—and recovery definitions—including absolute ALC, ARI, and relative change from EoRT ALC—underscore the lack of standardized criteria and highlight the need for further investigation to identify robust predictors of lymphocyte recovery.

This study aimed to: (1) evaluate the prognostic significance of lymphocyte recovery in patients with locally advanced ESCC receiving definitive RT; (2) compare the predictive value of recovery status at different post-RT time points; and (3) identify clinical and dosimetric predictors of lymphocyte recovery to develop a prediction model. These findings may facilitate individualized risk stratification and generate hypotheses for lymphocyte-sparing RT strategies, including the identification of patients at high risk of poor recovery and exploration of preliminary dose-volume constraints in lymphocyte-rich OARs that require prospective validation.

## 2. Materials and Methods

### 2.1. Patients

This is a single-center retrospective study. All patients with locally advanced ESCC who underwent definitive RT between January 2019 and December 2024 were consecutively screened for eligibility. Inclusion criteria were: (1) age ≥ 18 years; (2) Eastern Cooperative Oncology Group (ECOG) score ≤ 2; (3) pathologic confirmation of ESCC; (4) stage II–IVa according to the American Joint Committee on Cancer (AJCC) 8th edition, unresectable disease (e.g., cervical esophageal cancer, difficult to achieve R0 resection, contraindications to surgery, or high risk of surgery) or patient refusal of surgery; (5) initial definitive RT or definitive CRT; (6) radiation dose of at least 50 Gy; (7) full blood counts within 1 week of starting RT with serial measurements at 1 and 3 months after RT. Exclusion criteria were: (1) diseases that might affect lymphocyte count (e.g., hematologic malignancies, severe infection, or immunosuppression not caused by CRT); (2) prior surgery; (3) combination immunotherapy; (4) prior or concomitant malignancy; (5) pregnancy; (6) lack of clinical or follow-up information.

Patients’ clinical information was extracted from the electronic medical records of our institution. This retrospective analysis was approved by the Institutional Review Board of The Fourth Hospital of Hebei Medical University (protocol code 2024KS056).

### 2.2. Treatment and Follow-Up

All patients received intensity-modulated radiation therapy (IMRT), with or without chemotherapy. The gross tumor volume (GTV) included the primary tumor (GTV-t) and involved regional lymph nodes (GTV-nd) according to endoscopy, barium swallow, and computed tomography (CT). The clinical target volume (CTV) included CTV-t and CTV-nd. CTV-t was defined as the primary tumor plus a 2–3 cm expansion superiorly and inferiorly along the length of the esophagus and a 0.5–0.8 cm radial expansion. CTV-nd included GTV-nd and a 0.5 cm margin with or without the involved nodal regions, adjusted appropriately according to anatomical barriers. The planning target volume (PTV) included PTV-t and PTV-nd, defined as a 0.5 cm expansion around CTV-t and CTV-nd. Target volume delineation followed the National Health Commission of China guidelines for esophageal cancer radiotherapy (2022 edition) [16]. The prescribed radiation dose was 50–66 Gy for 95% PTV in 25–34 fractions (1.8–2.0 Gy per fraction) delivered once daily, five days per week. Dose limitations to OARs were: mean lung dose < 16 Gy, V_20_ < 30% and V_30_ < 20% for lungs; V_30_ < 40% and V_40_ < 30% for the heart; and maximum dose < 45 Gy for the spinal cord. Chemotherapy regimens were generally platinum-based doublets comprising: Paclitaxel plus platinum (TP): paclitaxel 175 mg/m^2^ d1 + cisplatin 75 mg/m^2^ d1, every 3 weeks; Fluorouracil plus platinum (FP): fluorouracil 750–1000 mg/m^2^ d1–4 + cisplatin 75–100 mg/m^2^ d1, every 4 weeks. All chemotherapy was delivered concurrently with RT.

Follow-up data were censored on 31 August 2025. Patients were followed up at 1 month post-treatment, every 3 months for the first 2 years, every 6 months until 5 years, and annually thereafter until death or last follow-up. Pretreatment staging evaluation included endoscopy with biopsy, barium swallow radiography, and contrast-enhanced chest/abdominal CT for all patients. Regular follow-up evaluations after treatment completion included contrast-enhanced cervical/thoracic/abdominal CT and barium swallow radiography for all patients. Gastroscopy was performed in patients with suspected local recurrence or stricture based on symptoms or imaging findings, as well as head magnetic resonance imaging (MRI) for suspected brain metastases and emission computed tomography (ECT) for suspected bone metastases.

### 2.3. Data Collection

Absolute lymphocyte count (ALC) values were collected before RT (Pre-RT), weekly during RT (W1–W5), at the end of RT (EoRT), at 1 month post-RT (P1), and at 3 months post-RT (P3). ΔALC_1_ was calculated as the difference between ALC at P1 and EoRT ALC, and ΔALC_3_ was defined as the difference between ALC at P3 and EoRT ALC, consistent with our previous study [15]. The lowest ALC during RT was identified as minALC and graded according to the Common Terminology Criteria for Adverse Events (CTCAE) version 5.0: Grade 1 (<1.1–0.8 × 10^9^/L), Grade 2 (<0.8–0.5 × 10^9^/L), Grade 3 (<0.5–0.2 × 10^9^/L), and Grade 4 (<0.2 × 10^9^/L).

### 2.4. Dose-Volume Parameters

The OARs included lungs, heart, sternum, ribs, thoracic vertebrae, aorta, and superior vena cava. Bone marrow was defined as the combined structure of the thoracic vertebrae, sternum, and ribs. The Pinnacle planning system 3.0 (Philips Healthcare, Fitchburg, MA, USA) was used to obtain mean doses and V_0.5_, V_1_, V_2_, V_3_, V_5_, V_10_, V_15_, V_20_, V_30_, and V_50_ for bones (sternum, ribs, and thoracic vertebrae) and V_5_, V_10_, V_15_, V_20_, V_25_, V_30_, V_35_, and V_40_ for other organs, determined by dose-volume histogram (DVH) analysis. V_x_ denotes the relative volume of specific organs receiving X Gy.

The effective dose to immune cells (EDIC) was calculated according to the formula proposed by Jin et al. [17]: EDIC = 0.12 × MLD + 0.08 × MHD + [0.45 + 0.35 × 0.85 × (of fractions/45)^1/2^] × ITDV/(61.8 × 10^3^). MLD, MHD and ITDV are the mean lung dose, mean heart dose and integral total dose volume. The coefficients reflect the relative blood volumes in these structures. EDIC estimates the radiation dose received by circulating lymphocytes during fractionated RT.

### 2.5. Statistical Analysis

Absolute lymphocyte count dynamics during RT were analyzed using the Friedman test for repeated measures across seven time points (pre-RT, weeks 1–5, and EoRT). Post hoc pairwise comparisons to pre-RT baseline were performed using the Wilcoxon signed-rank test with Bonferroni correction (significance threshold *p* < 0.0083, calculated as α/m = 0.05/6 for six comparisons). Lymphocyte recovery at P1 and P3 was compared to the EoRT nadir using the Wilcoxon signed-rank test with Bonferroni correction (significance threshold *p* < 0.025, calculated as α/m = 0.05/2 for two comparisons). Percentage decline was calculated individually for each patient as [(Pre-RT ALC − ALC at each time point)/Pre-RT ALC] × 100%. Overall survival (OS) and progression-free survival (PFS) were calculated from the start date of treatment to the date of death, disease progression, or the latest follow-up visit. Clinical baseline data between groups were compared using the chi-square test. The optimal cut-off points for ΔALC_1_ and ΔALC_3_ were determined by maximally selected log-rank statistics based on OS using the R package “maxstat” [18]. Kaplan–Meier analysis and log-rank test were used to compare differences in survival outcomes between groups. Survival analysis was assessed by univariate and multivariate Cox regression. Dose-volume parameters predicting lymphocyte recovery were transformed into binary variables by receiver operating characteristic (ROC) curve analysis, and the organ-specific parameter with the lowest *p*-value in univariate analysis was selected for multivariable adjustment. Univariable and multivariable logistic regression analyses were performed to correlate lymphocyte recovery with dose-volume parameters and other clinical factors. Variables with *p* < 0.05 on univariable Cox/logistic analysis were entered into multivariable analysis. To assess the effect of multicollinearity on regression, the variance inflation factor (VIF) was used with a cut-off of 5. A nomogram for predicting lymphocyte recovery probability was constructed based on the multivariate logistic regression model. Patients were assigned to training (*n* = 163) and validation (*n* = 70) cohorts using a 7:3 split. The area under the curve (AUC) of the ROC, calibration curve, and decision curve analysis (DCA) were used to evaluate the predictive performance and clinical utility of the nomogram. To reduce overfitting bias, bootstrap resampling was performed 1000 times. A two-tailed *p* < 0.05 was considered statistically significant. Data analysis was performed using SPSS version 23.0 (IBM SPSS Statistics, Armonk, NY, USA) and R software (version 4.3.3; R Foundation for Statistical Computing, Vienna, Austria), along with Zstats 1.0 (www.zstats.net, Zhejiang Chinese Medical University, Hangzhou, China).

## 3. Results

### 3.1. Patient Characteristics

A total of 233 eligible patients were enrolled, including 148 (63.5%) males and 85 (36.5%) females. The median age was 69 years. 58 (24.9%) patients had stage II disease, 104 (44.6%) had stage III, and 71 (30.5%) had stage IVa. The median tumor length was 5.0 cm. 166 (71.2%) patients received definitive CRT, and 67 (28.8%) received definitive RT. The median radiation dose was 60 Gy. Detailed clinical characteristics are listed in Table 1. No significant differences were observed between the training and validation cohorts.

### 3.2. ALC Dynamics and Recovery

**Figure 1:**
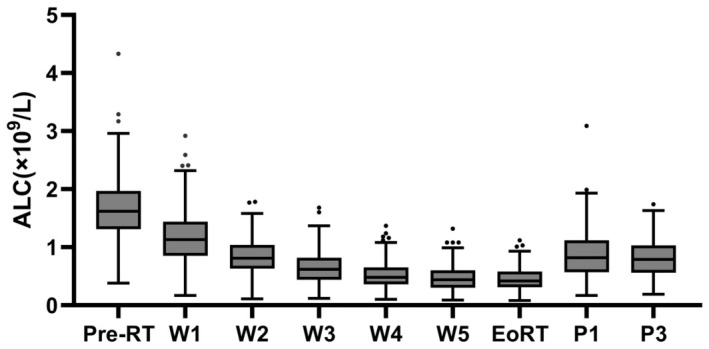
ALCs during and after RT. ALC declined progressively during RT, with significant reductions at all time points compared to pre-RT baseline (all *p* < 0.001, Wilcoxon signed-rank test with Bonferroni correction, significance threshold *p* < 0.0083). Post-RT recovery at P1 and P3 was also significant (both *p* < 0.001 vs. EoRT nadir, significance threshold *p* < 0.025). Annotation: Pre-RT: pre-RT, W1: week 1, W2: week 2, W3: week 3, W4: week 4, W5: week 5, EoRT: at the end of RT, P1: 1 month after RT finished, P3: 3 months after RT finished.

In the training cohort, the median pre-RT ALC was 1.62 × 10^9^/L. Only 33 (14.2%) patients showed pre-RT lymphopenia. ALC declined throughout RT (Friedman test, *p* < 0.001). The median ALCs (×10^9^/L) were 1.13, 0.81, 0.62, 0.48, 0.44, and 0.42 for week 1–5 during RT and at the end of RT, respectively (Figure 1). Compared to pre-RT baseline, ALC was significantly reduced at all measured time points during and at the end of RT (all *p* < 0.001, Wilcoxon signed-rank test with Bonferroni correction), with median percentage declines of 30.7%, 48.7%, 61.7%, 69.1%, 73.7%, and 73.3%, respectively. Following RT completion, ALC recovered gradually. The median ALCs were 0.82 × 10^9^/L and 0.79 × 10^9^/L at 1 month and 3 months post-RT, respectively. ALC recovered significantly at both P1 (*p* < 0.001) and P3 (*p* < 0.001) compared to the EoRT values. During RT, 34 (20.9%) patients showed grade 2 lymphopenia, 111 (68.1%) showed grade 3, and 18 (11.0%) showed grade 4.

### 3.3. Impact of Lymphocyte Recovery on Prognosis

The median follow-up time was 44 months. For the training cohort, the median OS was 26.4 months. The 1-year, 2-year, and 3-year OS rates were 75.8%, 52.0%, and 36.8%, respectively. The median PFS was 13.9 months. The 1-year, 2-year, and 3-year PFS rates were 53.8%, 38.0%, and 29.6%, respectively.

**Figure 2:**
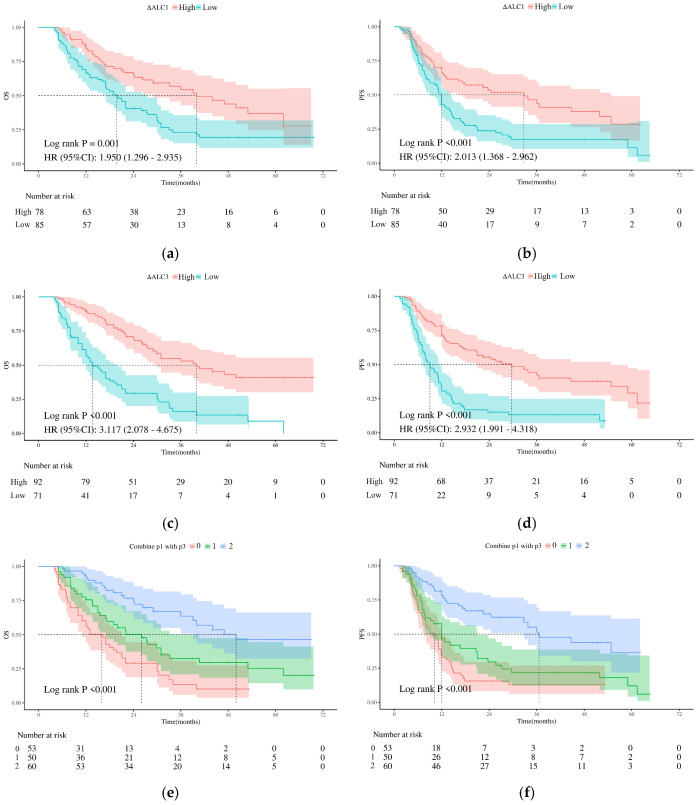
Kaplan–Meier curves of OS and PFS between different groups in the training cohort. Kaplan–Meier curves of OS (**a**) and PFS (**b**) curves between Group Low1 and Group High1; Kaplan–Meier curves of OS (**c**) and PFS (**d**) curves between Group Low and Group High; Kaplan–Meier curves of OS (**e**) and PFS (**f**) curves between Group 0, 1, and 2.

The optimal cut-off point for ΔALC_1_ was 0.41 × 10^9^/L. The median OS for Group Low1 (ΔALC_1_ ≤ 0.41 × 10^9^/L, *n* = 85) and Group High1 (ΔALC_1_ > 0.41 × 10^9^/L, *n* = 78) was 19.8 months versus 40.0 months (*p* = 0.001) (Figure 2a). The median PFS for Group Low1 and Group High1 was 11.9 months versus 32.8 months (*p* < 0.001) (Figure 2b).

The optimal cut-off point for ΔALC_3_ was 0.25 × 10^9^/L. The median OS for Group Low (ΔALC_3_ ≤ 0.25 × 10^9^/L, *n* = 71) and Group High (ΔALC_3_ > 0.25 × 10^9^/L, *n* = 92) was 13.7 months versus 40.0 months (*p* < 0.001) (Figure 2c). The median PFS for Group Low and Group High was 9.0 months versus 29.7 months (*p* < 0.001) (Figure 2d).

Patients were stratified into three groups based on lymphocyte recovery status at 1 month (P1) and 3 months (P3) post-RT: those who recovered at neither time point were assigned to Group 0, those who recovered at both time points were assigned to Group 2, and the remainder were assigned to Group 1. The median OS for Groups 0, 1, and 2 was 16.0, 26.0, and 50.0 months, respectively (*p* < 0.001) (Figure 2e). The median PFS for Groups 0, 1, and 2 was 10.2, 12.0, and 36.6 months, respectively (*p* < 0.001) (Figure 2f). Inadequate post-RT lymphocyte recovery was highly correlated with inferior survival.

The *p*-value for OS was lower when patients were grouped according to lymphocyte recovery status at 3 months post-RT. Consequently, subsequent analysis utilized lymphocyte recovery at 3 months post-RT.

Multivariate Cox regression revealed that ECOG score, T stage, tumor length, and lymphocyte recovery at 3 months post-RT were independent predictors of OS (Table 2). Higher lymphocyte recovery status was associated with a lower risk of death (HR 0.35, 95% CI 0.23–0.52, *p* < 0.001). T stage, tumor length, and lymphocyte recovery at 3 months post-RT were independent predictors of PFS. Higher lymphocyte recovery status was associated with a lower risk of disease progression (HR 0.37, 95% CI 0.25–0.54, *p* < 0.001).

To address potential immortal time bias, we performed a landmark analysis with P3 as the landmark time point. Lymphocyte recovery at P3 remained significantly associated with OS (*p* < 0.001) and PFS (*p* < 0.001) when OS and PFS were calculated from P3 (Supplementary Figure S1).

The optimal cut-off points for ΔALC_1_ and ΔALC_3_ were determined by maximally selected log-rank statistics in the training cohort. To assess potential overfitting, these thresholds were applied to the validation cohort (*n* = 70). When grouping patients by ΔALC_1_, the median OS for Group Low1 (*n* = 34) and Group High1 (*n* = 36) was 26.6 months versus not reached (*p* = 0.004) (Figure S2a). The median PFS for Group Low1 and Group High1 was 16.6 months versus 50.0 months (*p* = 0.034) (Figure S2b). When grouping patients by ΔALC_3_, the median OS for Group Low (*n* = 29) and Group High (*n* = 41) was 21.0 months versus not reached (*p* < 0.001) (Figure S2c). The median PFS for Group Low and Group High was 13.0 months versus not reached (*p* < 0.001) (Figure S2d).

To assess the generalizability of these findings, we applied the predefined ΔALC_1_ and ΔALC_3_ thresholds to the total cohort (*n* = 233). When grouping patients by ΔALC_1_, the median OS for Group Low1 (*n* = 119) and Group High1 (*n* = 114) was 22.0 months versus 50.0 months (*p* < 0.001) (Figure S3a). The median PFS for Group Low1 and Group High1 was 12.0 months versus 36.6 months (*p* < 0.001) (Figure S3b). When grouping patients by ΔALC_3_, the median OS for Group Low (*n* = 100) and Group High (*n* = 133) was 16.0 months versus not reached (*p* < 0.001) (Figure S3c). The median PFS for Group Low and Group High was 10.2 months versus 36.6 months (*p* < 0.001) (Figure S3d). The median OS for Groups 0 (*n* = 77), 1 (*n* = 65), and 2 (*n* = 91) was 17.0, 26.0, and not reached, respectively (*p* < 0.001) (Figure S3e). The median PFS for Groups 0, 1, and 2 was 11.1, 12.0, and 50.0 months, respectively (*p* < 0.001) (Figure S3f). After adjusting for sex, age, BMI, ECOG score, T stage, N stage, TNM stage, tumor location, tumor length, chemotherapy regimens, radiation dose, minALC, and preALC, multivariate Cox regression identified lymphocyte recovery at P3 as an independent predictor of OS (HR 0.29, 95% CI 0.20–0.42, *p* < 0.001) and PFS (HR 0.33, 95% CI 0.24–0.47, *p* < 0.001).

### 3.4. Construction and Validation of the Prediction Model

**Figure 3:**
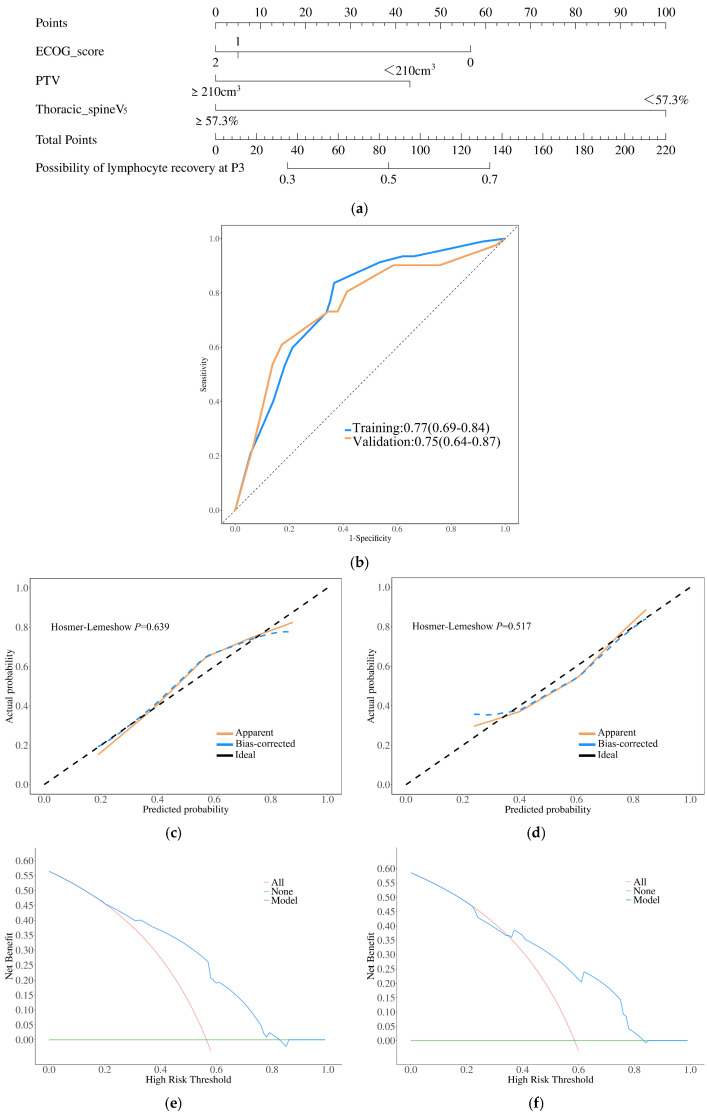
Nomogram and model performance metrics. Nomogram for predicting lymphocyte recovery 3 months post-RT (**a**), ROC curves of training and validation cohort (**b**), calibration curves of training (**c**) and validation cohort (**d**), DCA curves of training (**e**) and validation cohort (**f**).

Multivariate logistic analysis showed that ECOG score 1 (OR 0.41, 95% CI 0.19–0.89, *p* = 0.023) was independently associated with inadequate lymphocyte recovery at 3 months post-RT, while lower thoracic spine V_5_ (OR 5.55, 95% CI 2.71–11.36, *p* < 0.001) was independently associated with satisfactory lymphocyte recovery. Lower PTV (OR 2.10, 95% CI 1.00–4.41, *p* = 0.051) showed a borderline association with satisfactory lymphocyte recovery (Table 3). The complete univariate and multivariate logistic regression results for all evaluated DVH parameters are presented in Supplementary Table S1. Based on these factors, a nomogram was developed to predict the probability of lymphocyte recovery at 3 months post-RT (Figure 3a). The AUC values were 0.77 (95% CI 0.69–0.84) and 0.75 (95% CI 0.64–0.87) in the training and validation cohorts, respectively (Figure 3b). The calibration curves and DCA curves of the training and validation cohorts further indicated that the nomogram had good predictive performance and clinical utility (Figure 3c–f).

## 4. Discussion

To date, few reports have examined the impact of lymphocyte recovery on prognosis in ESCC. While our previous work described the prognostic significance of lymphocyte recovery, the present study extends these findings by exploring the feasibility of predicting lymphocyte recovery from pre-treatment variables, thereby generating hypotheses for future lymphocyte-sparing RT strategies. We combined lymphocyte recovery status at 1 month and 3 months post-RT to study its effect on survival outcomes. Analyses showed that post-RT lymphocyte recovery appears to be associated with improved OS and PFS after adjustment for conventional prognostic factors, though this observation requires confirmation in prospective, multicenter studies. Moreover, we established a prediction model for lymphocyte recovery after definitive RT. The ROC curves, calibration curves, and DCA curves showed good predictive performance. Through logistic analyses, we identified ECOG score and thoracic spine V_5_ as independent factors predicting lymphocyte recovery after RT; PTV showed a trend toward association, generating hypotheses regarding dose-volume constraints for lymphocyte-rich OARs, though prospective validation is required before any clinical implementation.

Many studies have focused on the adverse impact of radiation-induced lymphopenia during RT on the prognosis of esophageal cancer [9]. However, post-radiation lymphocyte recovery has not received sufficient attention. Deng et al. found that lymphocyte recovery 6–8 weeks after RT appeared unrelated to prognosis in esophageal cancer patients [13], whereas other studies found that lymphocyte recovery 6 months post-RT was associated with better survival outcomes in locally advanced ESCC [8,14]. The prognostic value of lymphocyte recovery after RT in esophageal cancer remains controversial, and the optimal time point for assessing lymphocyte recovery after RT has not been determined. Therefore, we conducted this study to further explore the prognostic value of post-RT lymphocyte recovery and compare the value of lymphocyte recovery at different time points after RT.

We compared the prognostic value of post-radiation lymphocyte recovery at different time points. Similar to our previous study [15], we observed that when grouping patients according to lymphocyte recovery status at P1, the survival differences were less significant than those observed when grouping patients according to lymphocyte recovery at P3. As shown in Figure 1, ALC values were unstable at 1 month post-RT and still showed fluctuation. A previous study also showed that lymphocyte recovery 6–8 weeks after RT was not associated with better prognosis in esophageal cancer [13]. Based on these findings, lymphocyte recovery at P3 appears to be a potentially more valuable factor than P1 for predicting survival in locally advanced ESCC. However, this observation requires validation in independent cohorts before definitive conclusions can be drawn. No consistent conclusion has been reached regarding the best time point to observe lymphocyte recovery status after RT. Further large-scale studies are warranted to identify the optimal time point for lymphocyte recovery assessment.

The optimal definition of lymphocyte recovery after RT remains debated. Alternative metrics include absolute ALC at specific time points [13], ALC recovery index (ratio to pre-RT ALC) [8,14], and CTCAE lymphopenia grade improvement. We selected ΔALC_3_ (ALC at P3 minus EoRT ALC) as the primary endpoint for several reasons. First, ΔALC_3_ captures the dynamic recovery process from treatment-induced lymphopenia, rather than a static measurement at a single time point. Second, EoRT ALC approximates the nadir for most patients after definitive RT, providing a clinically meaningful reference for assessing hematopoietic recovery capacity. Third, ΔALC_3_ is a continuous variable that preserves granular information compared with categorical metrics such as CTCAE grades. Fourth, our previous study [15] used ΔALC at P3 and demonstrated its prognostic value, supporting its selection in the current analysis. We acknowledge that no consensus exists on the optimal recovery metric and that comparative studies evaluating different definitions within the same cohort are warranted.

Unlike previous studies that only examined lymphocyte recovery at a single time point, we combined different time points after RT to observe dynamic changes in lymphocyte recovery and their impact on clinical outcomes. As expected, patients who recovered from RIL at both P1 and P3 had the best survival outcomes, and those who did not recover at either time point had the worst survival. Interestingly, patients who recovered at P1 or P3, representing an intermediate status, had intermediate survival outcomes. This may reflect an intermediate immune recovery capacity in these patients: stronger than those with persistent lymphopenia (Group 0), but weaker than those with sustained recovery (Group 2). This phenomenon suggests that although lymphocyte recovery status at P1 is unstable and its prognostic value is lower than P3, efforts should be made to promote lymphocyte recovery after RT completion as soon as possible. Even though some patients’ ALC values declined from P1 to P3, they might still benefit from transient high lymphocyte recovery status at P1 and had better survival than those who never recovered from RIL.

The prognostic value of lymphocyte nadir during RT identified in this study differs from that reported in other studies. Many studies have demonstrated that severe lymphopenia during RT was associated with poor survival in esophageal cancer [8,11,19]. However, this study did not observe a significant association between grade 3–4 RIL and prognosis. Several factors may contribute to this null finding. First, the statistical power to detect a significant association may have been limited, as the majority of patients (76.8%) experienced grade 3–4 lymphopenia, leaving a small comparison group (23.2% with grade 1–2). Second, the transient ALC nadir during RT cannot fully reflect patients’ potential anti-tumor immune status. In contrast, the dynamic changes in ALC after RT represent a more reliable biomarker for patients’ immune recovery and prognosis. Patients who experienced severe lymphopenia during RT might have satisfactory survival if they had a relatively high lymphocyte recovery status after RT. More prospective large-scale studies are warranted to compare the prognostic value of lymphopenia during RT and after RT completion.

We also developed a model predicting post-RT lymphocyte recovery, which has been less frequently investigated in previous studies. Numerous studies have developed models predicting RIL [10,12,20,21,22], most of which include factors such as radiation dose to the heart, lungs, spleen, and bone marrow. Correspondingly, the concept of lymphocyte-sparing RT has emerged. As the value of post-RT lymphocyte recovery in anti-tumor treatment gains increasing recognition, the role of lymphocyte-sparing RT in promoting lymphocyte recovery should also be thoroughly investigated. However, there is still disagreement on factors predicting lymphocyte recovery after RT. Therefore, we attempted to establish a model to predict lymphocyte recovery after RT and provide preliminary hypotheses for lymphocyte-sparing RT.

Factors related to radiation-related lymphocyte recovery have not reached a consensus. In multivariate logistic analyses, we identified ECOG score and thoracic spine V_5_ as independent predictive factors of lymphocyte recovery after RT; PTV showed a trend toward association. These findings are both consistent with and distinct from previous studies [8,14]. A higher ECOG score indicates poorer general condition and nutritional status, which in turn leads to impaired hematopoietic function. Previous studies have not attached importance to this factor; therefore, we suggest that clinicians prioritize the patient’s general condition and implement proactive supportive care before initiating treatment, rather than solely focusing on anti-tumor therapy and immune-boosting interventions. We also found that larger PTV showed a borderline association with lower lymphocyte recovery status, which is consistent with previous studies [8,23]. Large PTV usually indicates that lymphocyte-rich OARs receive a relatively large volume of irradiation at a certain radiation dose. It is noteworthy that PTV did not reach conventional statistical significance (*p* = 0.051) in multivariate logistic analysis, despite a point estimate suggesting a clinically relevant effect (OR 2.10). The 95% confidence interval (1.00–4.41) included the null value at its lower bound, indicating uncertainty that warrants cautious interpretation. Future studies with larger sample sizes are needed to clarify whether PTV is a true independent predictor of lymphocyte recovery.

The effect of radiation dose to bone marrow on lymphocyte recovery warrants attention. Consistent with previous research by Cheung et al. [8], we studied the hematopoietic bone marrow in the chest separately to examine its effect on lymphocyte recovery, rather than treating it as a single entity. We found that radiation dose to the thoracic spine was a stronger indicator of lymphocyte recovery than radiation dose to all bone marrow in the chest. Because not all axial bones are equally important in hematopoiesis, it might be more realistic to treat different bones separately rather than as a whole in clinical practice. When radiation physicists develop RT plans requiring bone marrow protection but are unable to fully account for all hematopoietic bones, perhaps they should prioritize protecting the thoracic spine.

However, no consensus has been reached on specific bone marrow dose constraints. Cheung et al. reported thoracic spine V_35_ as a predictor of lymphocyte recovery in esophageal cancer with a robust cut-off at 25% [8]. In contrast, we observed that thoracic spine V_5_ was an independent predictor of lymphocyte recovery with a cut-off at 57.3%, which indicates that the volume of the thoracic spine exposed to low-dose radiation also warrants attention. Similarly, Tseng et al. also found that a larger relative volume of bone marrow receiving ≥5 Gy was correlated with a higher risk of insufficient lymphocyte recovery [7], which confirms our speculation. These findings raise the hypothesis that low-dose radiation volume to the thoracic spine may influence lymphocyte recovery. However, whether reducing thoracic spine V_5_ in clinical practice will improve lymphocyte recovery or survival remains unproven and requires prospective validation.

EDIC has been shown to be correlated with RIL in esophageal cancer [7,10,11,24]. However, neither our study nor others have found a significant correlation between EDIC and lymphocyte recovery after RT completion [8,14]. This might be because EDIC is calculated based on mean doses to the heart, lungs, and whole body, and RT fractions. It primarily reflects the radiation dose received by circulating blood, indicating the immediate lethal effect of radiation on lymphocytes in peripheral blood. However, this does not affect the recovery of lymphocytes through hematopoiesis by the bone marrow after RT completion.

Questions remain as to whether lymphocyte recovery can be promoted by reducing the dose to the OARs described in the current study, and whether the promotion of lymphocyte recovery can translate into a corresponding improvement in survival outcomes. Future prospective trials are required to validate the impact of lymphocyte-sparing OAR constraints on lymphocyte recovery and outcome.

Lymphocyte recovery after RT is influenced by multiple factors beyond radiation dose, including nutritional status, systemic inflammation, treatment interruption, chemotherapy intensity, tumor progression, and general frailty. Our retrospective design did not systematically capture these variables, and they were therefore not included in the prediction model. This represents an important limitation, as the observed association between thoracic spine V_5_ and lymphocyte recovery may be partially confounded by unmeasured patient-related factors. Future prospective studies should incorporate these factors to better elucidate the independent contribution of dosimetric parameters.

This study has limitations. First, the retrospective design and absence of external validation may affect generalizability; although bootstrap internal validation with 1000 resamples was performed, prospective multicenter validation is essential before clinical application. Second, treatment heterogeneity existed: 28.8% of patients received RT alone, while 71.2% received CRT, which may influence lymphocyte recovery and survival. Third, immune functional markers (e.g., T-cell proliferation, cytokine profiles, cytotoxic activity) and lymphocyte subtypes were not assessed, limiting mechanistic insights into the potential immunomodulatory effects of lymphocyte recovery. Fourth, inflammatory markers (e.g., neutrophil-to-lymphocyte ratio, platelet-to-lymphocyte ratio, C-reactive protein) were not systematically assessed. These markers could provide additional context for systemic immune status and help better interpret the prognostic value of lymphocyte recovery. Future studies should incorporate comprehensive inflammatory profiling alongside ALC dynamics. Fifth, we acknowledge that the cut-off points for ΔALC_1_ and ΔALC_3_ were derived from exploratory analyses using maximally selected log-rank statistics. Although these thresholds were validated in our internal validation cohort, they remain exploratory and require prospective external validation in independent multicenter cohorts before clinical application. Further research is needed to explore the impact of radiation dose to lymphocyte-rich OARs on different lymphocyte subsets, thereby deepening our understanding of the mechanisms underlying the OARs-lymphocyte recovery-prognosis axis.

Despite these limitations, our study combined lymphocyte recovery status at 1 month and 3 months post-RT to predict prognosis in locally advanced ESCC and built a prediction model for post-RT lymphocyte recovery. This preliminary, internally validated model may serve as a foundation for future external validation and potential clinical implementation.

## 5. Conclusions

Superior lymphocyte recovery after definitive RT appears to be independently associated with improved OS and PFS in locally advanced ESCC, though this observation requires confirmation in prospective, multicenter studies. ECOG performance status and thoracic spine V_5_ were identified as predictors of lymphocyte recovery, and PTV showed a trend toward association. The proposed prediction model demonstrates preliminary, internally validated discrimination and requires external validation before clinical implementation.

## Figures and Tables

**Table 1:** Characteristics of patients.

Variables	Total (*n* = 233)	Training Cohort (*n* = 163)	Validation Cohort (*n* = 70)	Chi-Square Value (χ^2^)	*p*
Sex, *n* (%)				1.06	0.304
Male	148 (63.52)	107 (65.64)	41 (58.57)		
Female	85 (36.48)	56 (34.36)	29 (41.43)		
Age, *n* (%)				0.00	0.957
<65	66 (28.33)	46 (28.22)	20 (28.57)		
≥65	167 (71.67)	117 (71.78)	50 (71.43)		
BMI, *n* (%)				0.04	0.982
<18.5	22 (9.44)	15 (9.20)	7 (10.00)		
18.5–24.9	151 (64.81)	106 (65.03)	45 (64.29)		
>24.9	60 (25.75)	42 (25.77)	18 (25.71)		
ECOG score, *n* (%)				0.31	0.856
0	105 (45.06)	73 (44.79)	32 (45.71)		
1	97 (41.63)	67 (41.10)	30 (42.86)		
2	31 (13.30)	23 (14.11)	8 (11.43)		
T stage, *n* (%)				1.00	0.607
2	86 (36.91)	58 (35.58)	28 (40.00)		
3	128 (54.94)	90 (55.21)	38 (54.29)		
4	19 (8.15)	15 (9.20)	4 (5.71)		
N stage, *n* (%)				7.06	0.070
0	28 (12.02)	19 (11.66)	9 (12.86)		
1	52 (22.32)	29 (17.79)	23 (32.86)		
2	95 (40.77)	72 (44.17)	23 (32.86)		
3	58 (24.89)	43 (26.38)	15 (21.43)		
TNM stage ^1^, *n* (%)				4.77	0.092
II	58 (24.89)	34 (20.86)	24 (34.29)		
III	104 (44.64)	76 (46.63)	28 (40.00)		
IVa	71 (30.47)	53 (32.52)	18 (25.71)		
Tumor location, *n* (%)				2.56	0.278
Upper	77 (33.05)	57 (34.97)	20 (28.57)		
Middle	79 (33.91)	50 (30.67)	29 (41.43)		
Lower	77 (33.05)	56 (34.36)	21 (30.00)		
Tumor length, *n* (%)				0.67	0.413
≤5 cm	126 (54.08)	91 (55.83)	35 (50.00)		
>5 cm	107 (45.92)	72 (44.17)	35 (50.00)		
Chemotherapy, *n* (%)				0.98	0.323
Yes	166 (71.24)	113 (69.33)	53 (75.71)		
No	67 (28.76)	50 (30.67)	17 (24.29)		
Radiation dose ^2^, *n* (%)				0.02	0.876
<60 Gy	128 (54.94)	89 (54.60)	39 (55.71)		
≥60 Gy	105 (45.06)	74 (45.40)	31 (44.29)		
minALC ^3^, *n* (%)				1.64	0.201
G1–2	54 (23.18)	34 (20.86)	20 (28.57)		
G3–4	179 (76.82)	129 (79.14)	50 (71.43)		
preALC, *n* (%)				2.80	0.094
<1.1 × 10^9^/L	33 (14.16)	19 (11.66)	14 (20.00)		
≥1.1 × 10^9^/L	200 (85.84)	144 (88.34)	56 (80.00)		

Acronyms: BMI, Body Mass Index; ECOG, Eastern Cooperative Oncology Group; ALC, Absolute lymphocyte count. ^1^ According to AJCC 8th. ^2^ Radiation dose refers to the prescribed dose to the planning target volume (primary tumor and involved nodal regions). ^3^ The lowest ALC during RT was identified as minALC and graded according to the Common Terminology Criteria for Adverse Events version 5.0.

**Table 2:** Univariate and multivariate COX analysis for OS and PFS.

Variables	OS				PFS			
	Univariate Analyses		Multivariate Analysis		Univariate Analyses		Multivariate Analysis	
	*p*	HR (95% CI)	*p*	HR (95% CI)	*p*	HR (95% CI)	*p*	HR (95% CI)
Sex								
Male		1.00				1.00		
Female	0.067	0.66 (0.42~ 1.03)			0.082	0.69 (0.46~1.05)		
Age								
<65		1.00				1.00		
≥65	0.497	1.17 (0.75~1.82)			0.507	0.87 (0.58~1.31)		
BMI								
<18.5		1.00				1.00		
18.5–24.9	0.977	1.01 (0.50~2.03)			0.666	1.16 (0.60~2.24)		
>24.9	0.276	0.65 (0.30~1.42)			0.356	0.70 (0.33~1.49)		
ECOG score								
0		1.00				1.00		
1	0.053	1.53 (0.99~2.37)	1.000	1.00 (0.63~1.60)	0.017	1.63 (1.09~2.44)		
2	0.006	2.28 (1.27~4.12)	0.017	2.08 (1.14~3.80)	0.125	1.59 (0.88~2.87)		
T stage								
2		1.00		1.00		1.00		1.00
3	0.008	1.84 (1.17~2.89)	0.049	1.58 (1.01~2.50)	0.008	1.80 (1.17~2.78)	0.036	1.59 (1.03~2.46)
4	<0.001	3.28 (1.69~6.36)	0.015	2.44 (1.19~5.02)	0.003	2.62 (1.39~4.95)	0.117	1.69 (0.88~3.24)
N stage								
0		1.00				1.00		
1	0.998	1.00 (0.45~2.24)			0.771	0.90 (0.42~1.89)		
2	0.282	1.46 (0.73~2.92)			0.266	1.45 (0.75~2.78)		
3	0.073	1.95 (0.94~4.05)			0.053	1.98 (0.99~3.95)		
TNM stage ^1^								
II		1.00				1.00		
III	0.137	1.54 (0.87~2.73)			0.064	1.68 (0.97~2.91)		
IVa	0.006	2.32 (1.28~4.20)			0.002	2.53 (1.42~4.49)		
Tumor location								
Upper		1.00				1.00		
Middle	0.376	1.26 (0.75~2.12)			0.721	1.09 (0.67~1.77)		
Lower	0.036	1.66 (1.03~2.66)			0.085	1.48 (0.95~2.31)		
Tumor length,								
≤5 cm		1.00		1.00		1.00		1.00
>5 cm	0.004	1.79 (1.20~2.66)	0.019	1.64 (1.09~2.48)	<0.001	2.04 (1.40~2.97)	0.002	1.83 (1.25~2.69)
Chemotherapy								
Yes		1.00				1.00		
No	0.204	0.76 (0.50~1.16)			0.432	0.85 (0.57~1.27)		
Chemotherapy regimens								
TP		1.00				1.00		
Others	0.996	1.00 (0.54~1.85)			0.439	1.24 (0.72~2.16)		
None	0.233	1.32 (0.85~2.05)			0.340	1.23 (0.81~1.87)		
Radiation dose								
<60 Gy		1.00				1.00		
≥60 Gy	0.649	0.91 (0.61~1.36)			0.815	0.96 (0.66~1.39)		
minALC ^2^								
G3–4		1.00				1.00		
G1–2	0.330	0.77 (0.46~1.30)			0.320	0.78 (0.49~1.26)		
preALC								
<1.1 × 10^9^/L		1.00				1.00		
≥1.1 × 10^9^/L	0.446	0.80 (0.44~1.43)			0.282	0.74 (0.43~1.28)		
Lymphocyte recovery at 3 months post-RT								
No		1.00		1.00		1.00		1.00
Yes	<0.001	0.32 (0.21~0.48)	<0.001	0.35 (0.23~0.52)	<0.001	0.34 (0.23~0.50)	<0.001	0.37 (0.25~0.54)

Acronyms: BMI, Body Mass Index; ECOG, Eastern Cooperative Oncology Group; ALC, Absolute lymphocyte count. ^1^ According to AJCC 8th. ^2^ The lowest ALC during RT was identified as minALC and graded according to the Common Terminology Criteria for Adverse Events version 5.0.

**Table 3:** Factors related to lymphocyte recovery.

Factors	Multivariate Analysis		Variance Inflation Factor
	*p*	OR (95% CI)	
ECOG score			1.007
0		1.00	
1	0.023	0.41 (0.19~0.89)	
2	0.078	0.38 (0.13~1.11)	
PTV			1.048
≥210 cm^3^		1.00	
<210 cm^3^	0.051	2.10 (1.00~4.41)	
Thoracic spine V_5_^1^			1.055
≥57.3%		1.00	
<57.3%	<0.001	5.55 (2.71~11.36)	

Acronyms: ECOG, Eastern Cooperative Oncology Group; PTV, Planning Target Volume. ^1^ V_x_ denotes the relative volume of specific organs receiving X Gy.

## Data Availability

The data presented in this study are available on request from the corresponding author.

## Supplementary Materials

The following supporting information can be downloaded at: https://www.mdpi.com/article/10.3390/curroncol33060374/s1, Figure S1: Kaplan–Meier curves of OS (P3) (a) and PFS (P3) (b) between Group Low and Group High; Figure S2: Kaplan–Meier curves of OS and PFS between different groups in the validation cohort. Kaplan–Meier curves of OS (a) and PFS (b) curves between Group Low1 and Group High1; Kaplan–Meier curves of OS (c) and PFS (d) curves between Group Low and Group High; Figure S3: Kaplan–Meier curves of OS and PFS between different groups in the total cohort. Kaplan–Meier curves of OS (a) and PFS (b) curves between Group Low1 and Group High1; Kaplan–Meier curves of OS (c) and PFS (d) curves between Group Low and Group High; Kaplan–Meier curves of OS (e) and PFS (f) curves between Group 0, 1, and 2; Table S1: Factors related to lymphocyte recovery: univariate and multivariate binary logistic regression analysis.

## Abbreviations

The following abbreviations are used in this manuscript:

ALC	Absolute lymphocyte count
AUC	Area under the curve
BMI	Body mass index
CTCAE	Common Terminology Criteria for Adverse Events
CTV	Clinical target volume
CT	Computed tomography
CRT	Chemoradiotherapy
DCA	Decision curve analysis
DVH	Dose-volume histogram
ECOG	Eastern Cooperative Oncology Group
EDIC	Effective dose to immune cells
ECT	Emission computed tomography
ESCC	Esophageal squamous cell carcinoma
FP	Fluorouracil plus platinum
GTV	Gross tumor volume
IMRT	Intensity-modulated radiation therapy
MRI	Magnetic resonance imaging
OAR	Organ at risk
OS	Overall survival
PFS	Progression-free survival
PTV	Planning target volume
PET	Positron emission tomography
RIL	Radiation-induced lymphopenia
RT	Radiotherapy
ROC	Receiver operating characteristic curve
TP	Paclitaxel plus platinum
VIF	Variance inflation factor
