# Supplementary material for: Predicting Post-Radiotherapy Lymphocyte Recovery for Individualized Risk Stratification in Locally Advanced Esophageal Squamous Cell Carcinoma
Source: Curr Oncol. 2026 Jun 22;33(6):374. doi: 10.3390/curroncol33060374 (PMC13297973; doi:10.3390/curroncol33060374)
Supplement: Supplementary file 1 [file curroncol-33-00374-s001.zip › curroncol-4265261-supplementary.pdf]

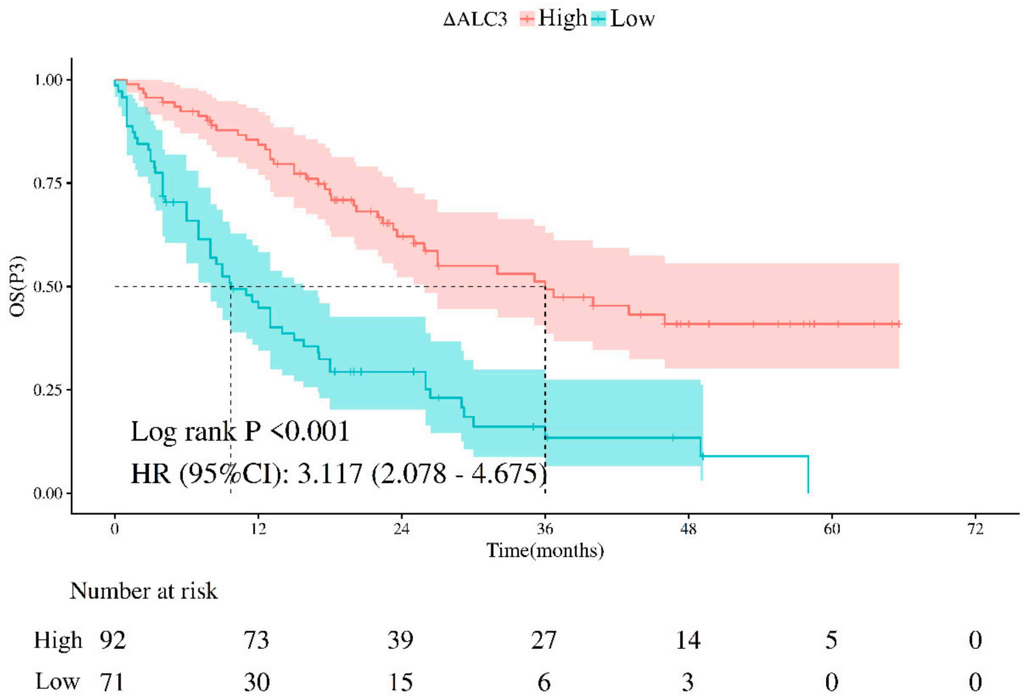

(**a**)

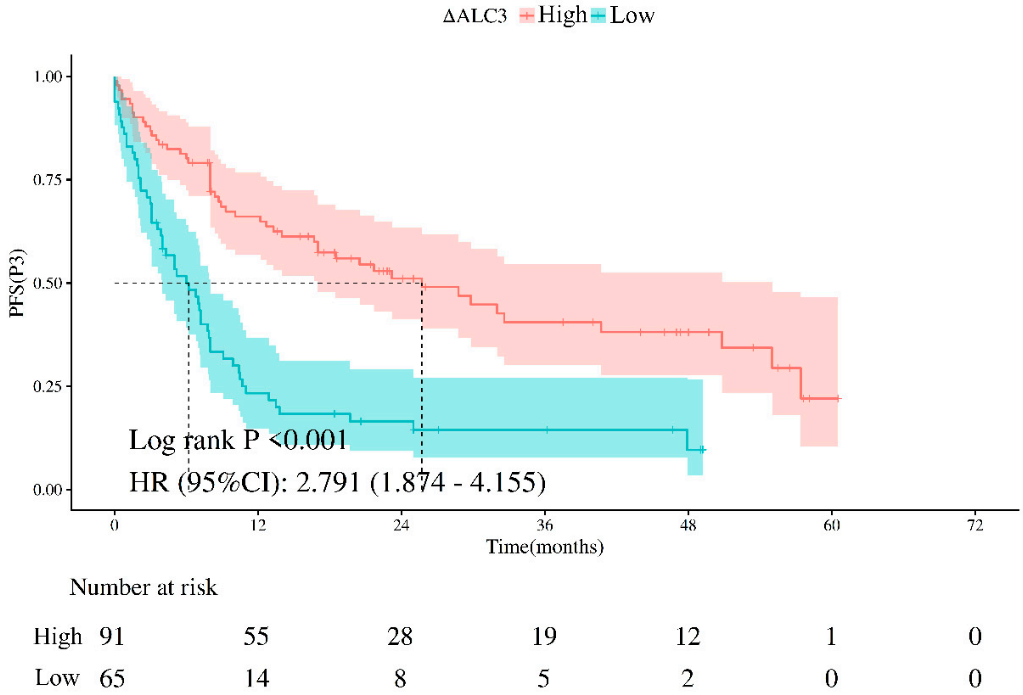

(**b**)

Figure S1. Kaplan–Meier curves of OS (P3) (**a**) and PFS (P3) (**b**) between Group Low and Group High.

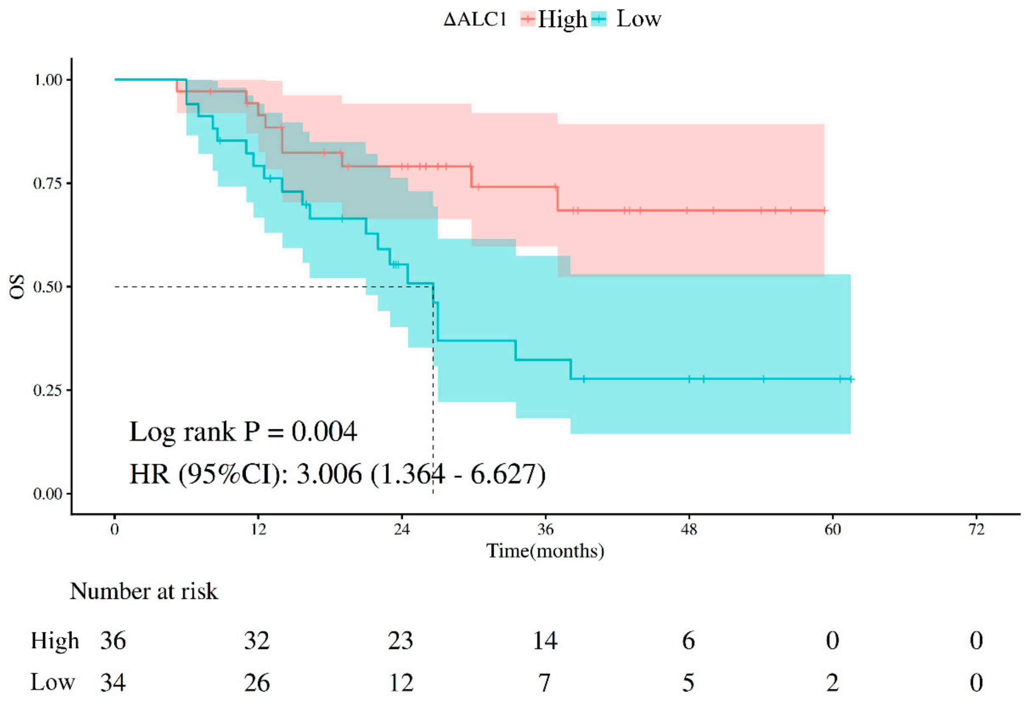
ΔALC1 High Low
OS
1.00
0.75
0.50
0.25
0.00
Log rank P = 0.004
HR (95%CI): 3.006 (1.364 - 6.627)
0 12 24 36 48 60 72
Time(months)
Number at risk
High 36 32 23 14 6 0 0
Low 34 26 12 7 5 2 0

(**a**)

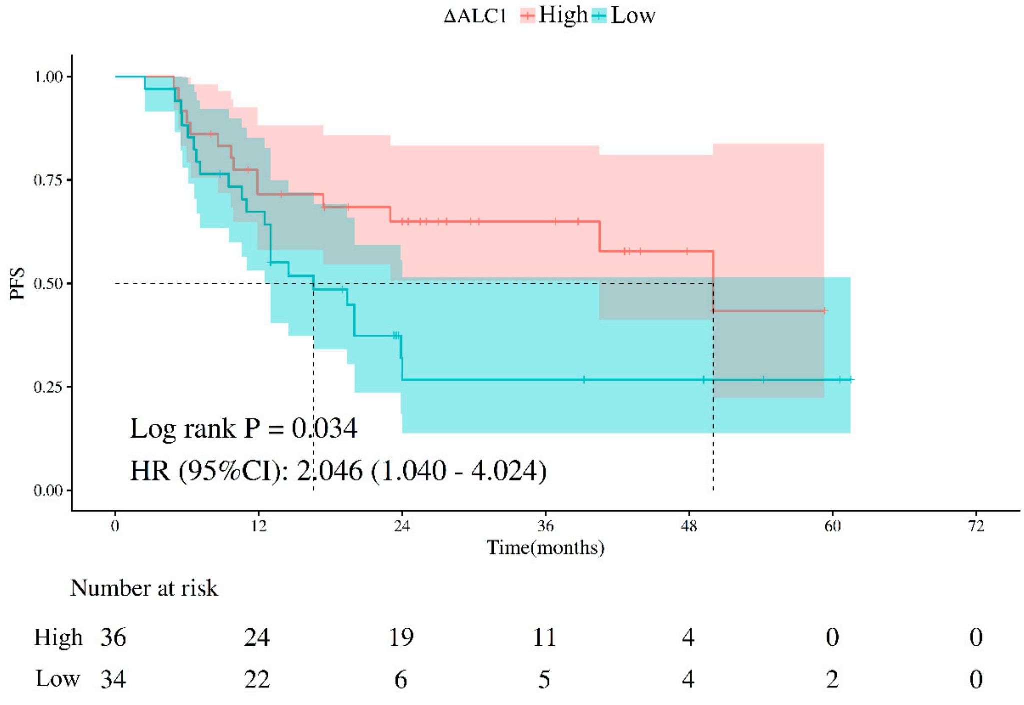
ΔALC1 High Low
PFS
1.00
0.75
0.50
0.25
0.00
Log rank P = 0.034
HR (95%CI): 2.046 (1.040 - 4.024)
0 12 24 36 48 60 72
Time(months)
Number at risk
High 36 24 19 11 4 0 0
Low 34 22 6 5 4 2 0

(**b**)

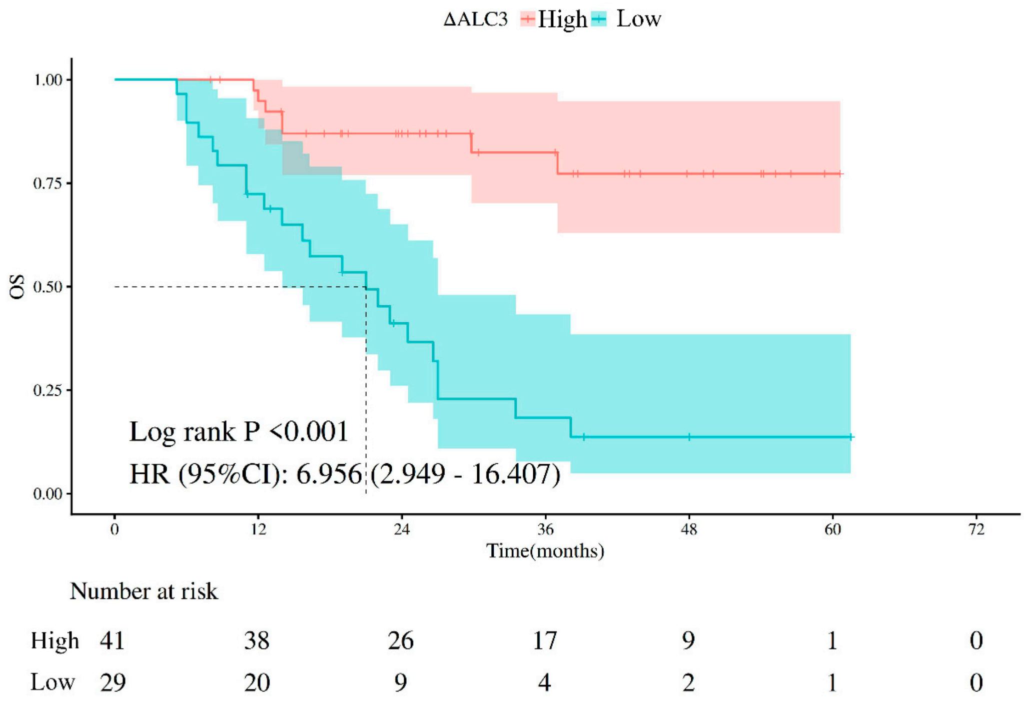

(**c**)

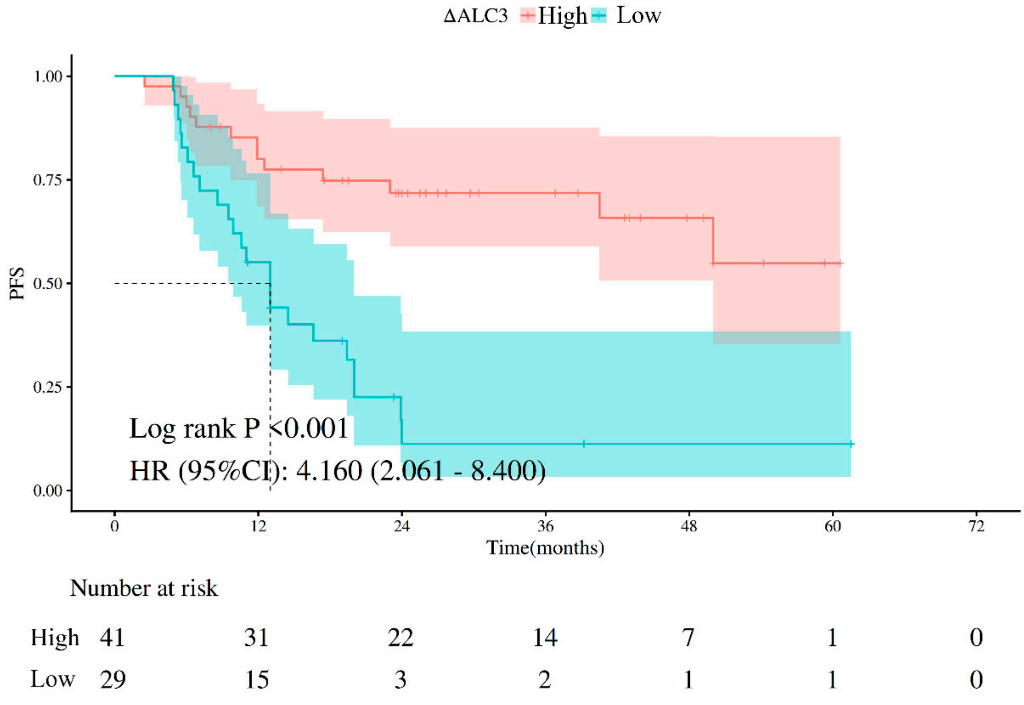

(**d**)

Figure S2. Kaplan–Meier curves of OS and PFS between different groups in the validation cohort. Kaplan–Meier curves of OS (**a**) and PFS (**b**) curves between Group Low1 and Group High1; Kaplan–Meier curves of OS (**c**) and PFS (**d**) curves between Group Low and Group High.

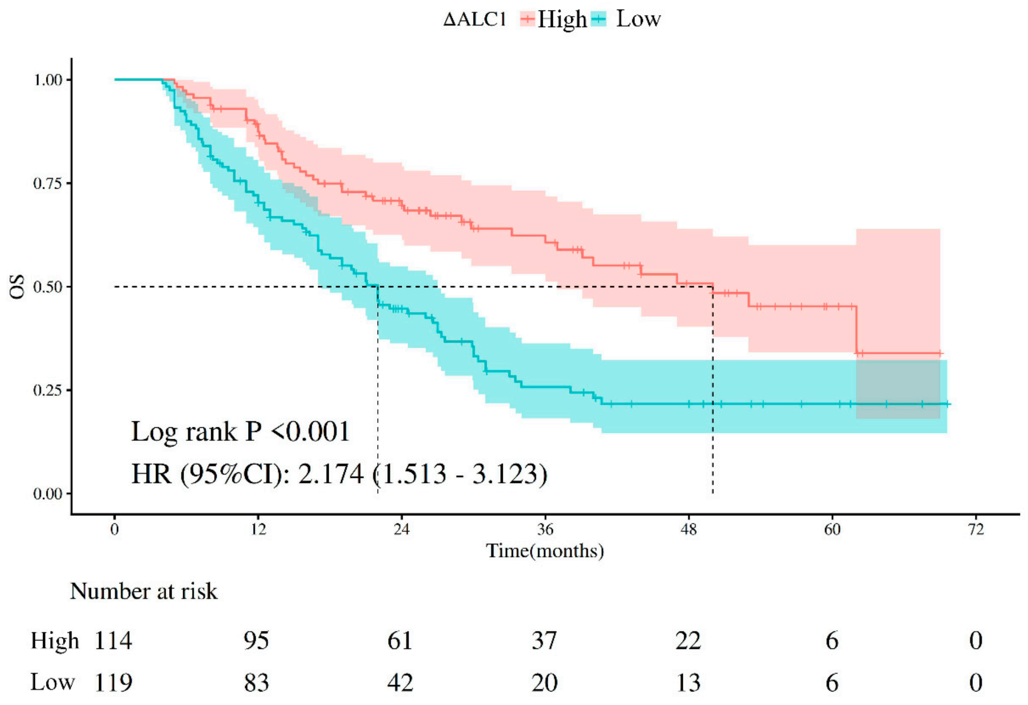
ΔALC1 High Low
1.00
0.75
0.50
0.25
0.00
OS
Log rank P <0.001
HR (95%CI): 2.174 (1.513 - 3.123)
0
12
24
36
48
60
72
Time(months)
Number at risk
High 114 95 61 37 22 6 0
Low 119 83 42 20 13 6 0

(**a**)

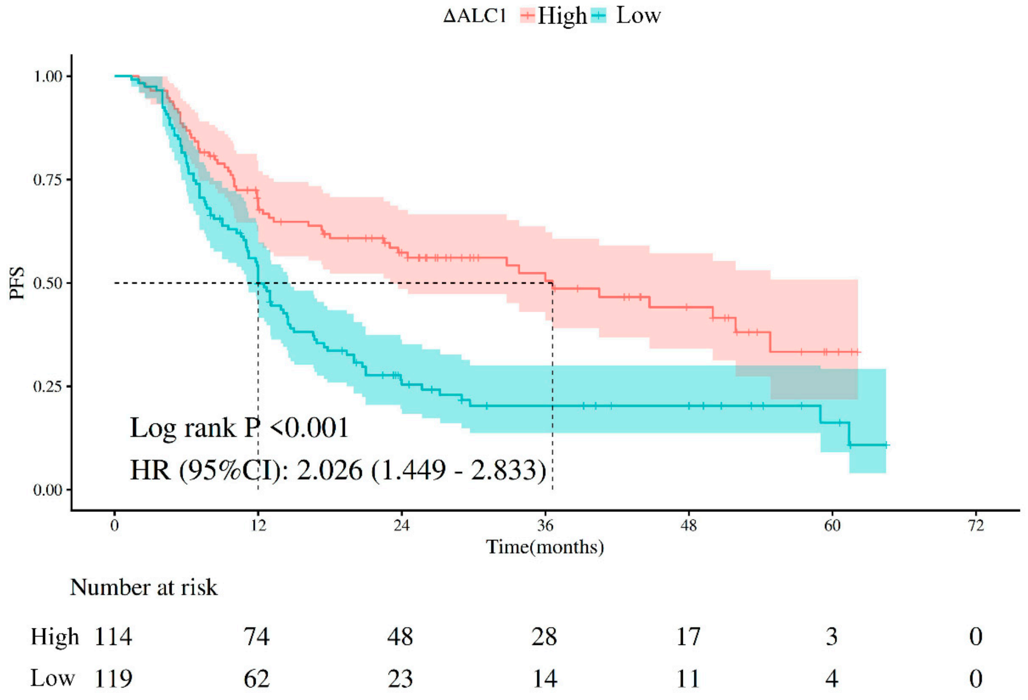
ΔALC1 High Low
1.00
0.75
0.50
0.25
0.00
PFS
Log rank P <0.001
HR (95%CI): 2.026 (1.449 - 2.833)
0
12
24
36
48
60
72
Time(months)
Number at risk
High 114 74 48 28 17 3 0
Low 119 62 23 14 11 4 0

(**b**)

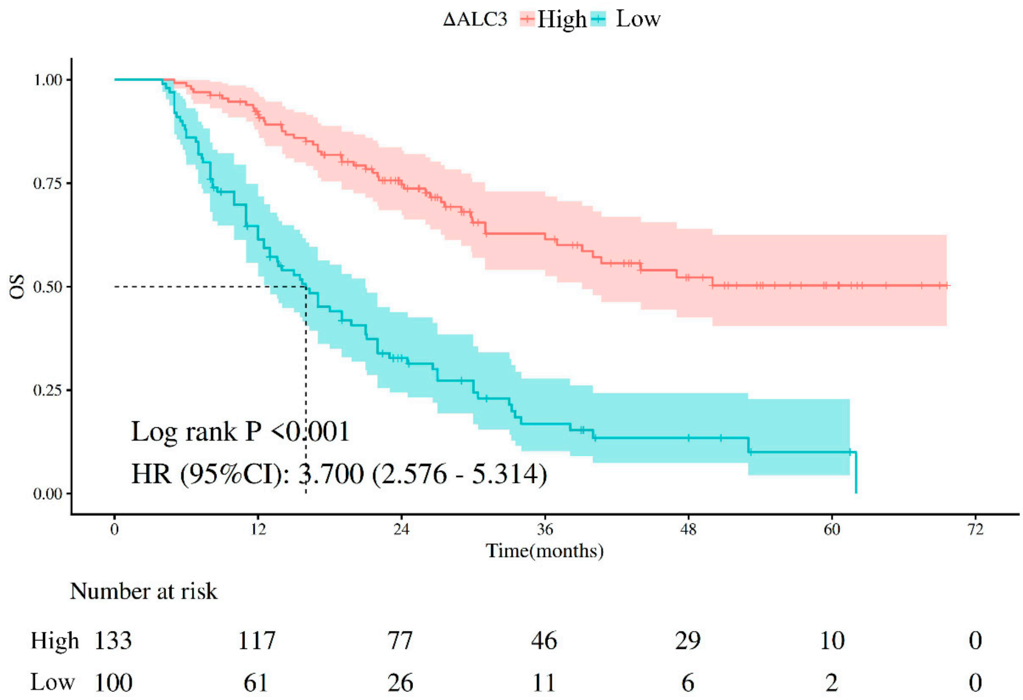
ΔALC3 High Low
1.00
0.75
0.50
0.25
0.00
OS
Log rank P <0.001
HR (95%CI): 3.700 (2.576 - 5.314)
0 12 24 36 48 60 72
Time(months)
Number at risk
High 133 117 77 46 29 10 0
Low 100 61 26 11 6 2 0

(c)

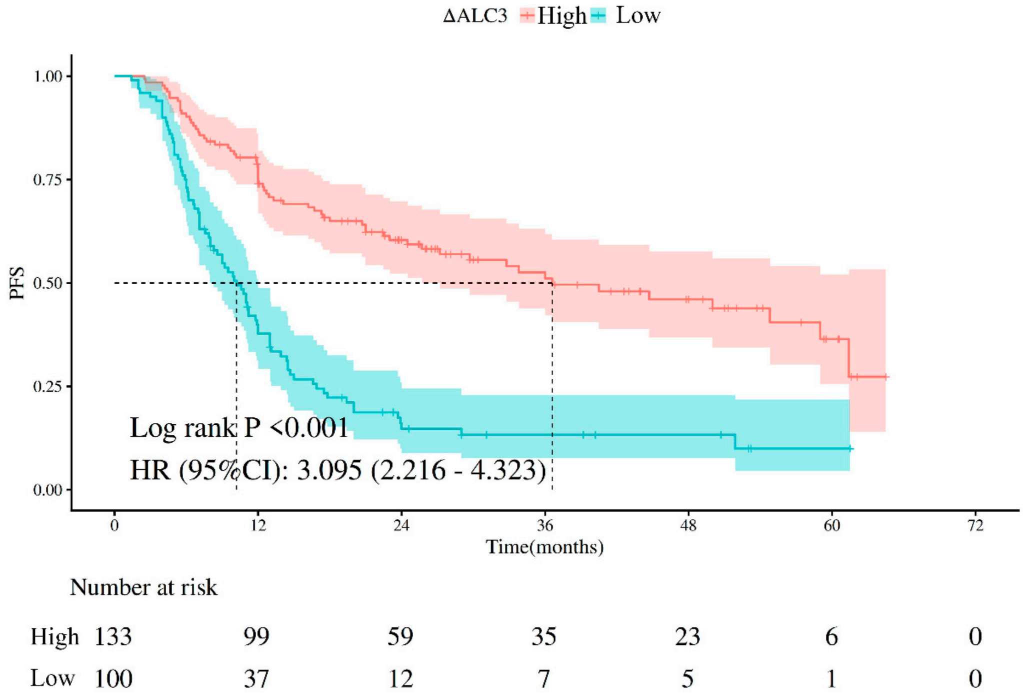
ΔALC3 High Low
1.00
0.75
0.50
0.25
0.00
PFS
Log rank P <0.001
HR (95%CI): 3.095 (2.216 - 4.323)
0 12 24 36 48 60 72
Time(months)
Number at risk
High 133 99 59 35 23 6 0
Low 100 37 12 7 5 1 0

(d)

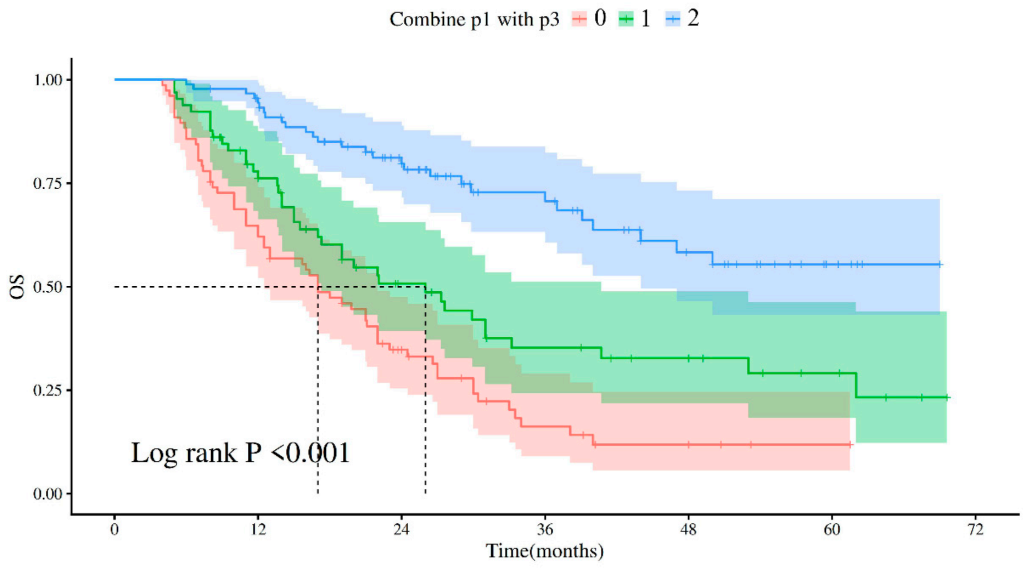

Number at risk

0	77	49	22	8	4	1	0
1	65	46	24	15	11	6	0
2	91	83	57	34	20	5	0

(**e**)

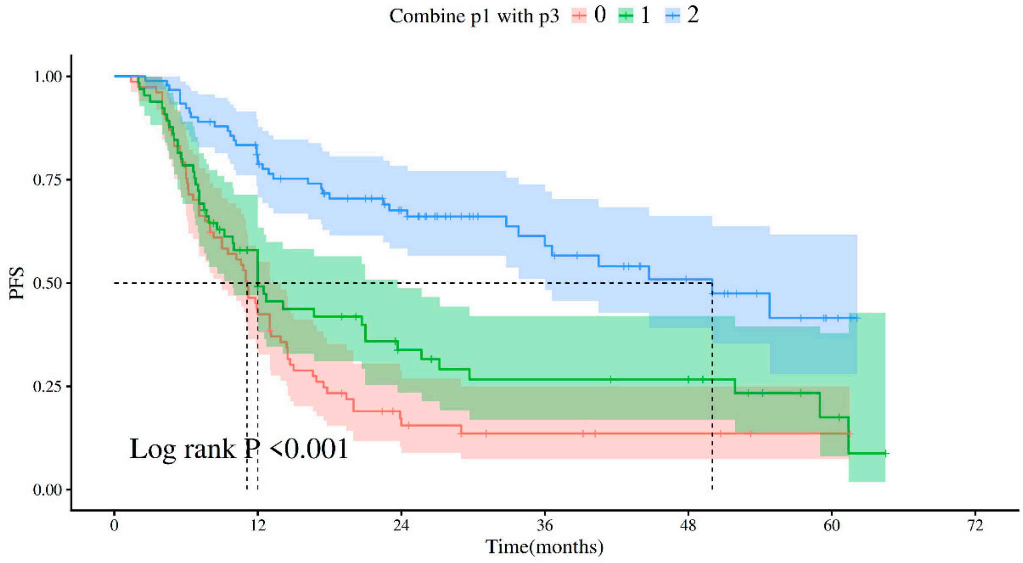

Number at risk

0	77	33	10	5	3	1	0
1	65	33	15	11	10	3	0
2	91	70	46	26	15	3	0

(**f**)

Figure S3. Kaplan–Meier curves of OS and PFS between different groups in the total cohort. Kaplan–Meier curves of OS (**a**) and PFS (**b**) curves between Group Low1 and Group High1; Kaplan–Meier curves of OS (**c**)

and PFS (**d**) curves between Group Low and Group High; Kaplan–Meier curves of OS (**e**) and PFS (**f**) curves between Group 0, 1, and 2.

Table S1. Factors related to lymphocyte recovery: univariate and multivariate binary logistic regression analysis.

Factors	Univariate analyses		Multivariate analysis		Variance inflation factor
	P	**OR (95%CI)**	***P***	**OR (95%CI)**	
Sex					
Male		1.00			
Female	0.427	1.31 (0.68 ~ 2.52)			
Age					
＜65		1.00			
≥65	0.475	0.78 (0.39 ~ 1.56)			
BMI					
＜18.5		1.00			
18.5-24.9	0.557	0.72 (0.24 ~ 2.16)			
＞24.9	0.643	1.33 (0.40 ~ 4.50)			
ECOG score					1.007
0		1.00		1.00	
1	0.008	0.40 (0.20 ~ 0.79)	0.023	0.41 (0.19 ~ 0.89)	
2	0.077	0.42 (0.16 ~ 1.10)	0.078	0.38 (0.13 ~ 1.11)	
T stage					
2		1.00			
3	0.261	0.68 (0.34 ~ 1.34)			
4	0.102	0.38 (0.12 ~ 1.21)			
N stage					
0		1.00			
1	0.683	1.27 (0.40 ~ 4.09)			
2	0.504	1.41 (0.51 ~ 3.91)			
3	0.783	0.86 (0.29 ~ 2.53)			
TNM stage[1]					
Ⅱ		1.00			
Ⅲ	0.556	1.28 (0.56 ~ 2.91)			
Ⅳa	0.535	0.76 (0.32 ~ 1.81)			
Tumor location					
Upper		1.00			
Middle	0.486	0.75 (0.34 ~ 1.67)			

Lower	0.002	0.30 (0.14 ~ 0.65)			
Tumor length					
≤5cm		1.00			
>5cm	0.141	0.63 (0.33 ~ 1.17)			
Chemotherapy					
No		1.00			
Yes	0.676	1.15 (0.59 ~ 2.25)			
Chemotherapy regimens					
TP		1.00			
Others	0.312	0.61 (0.24 ~ 1.59)			
None	0.506	0.79 (0.39 ~ 1.58)			
Radiation dose					
<60Gy		1.00			
≥60Gy	0.306	1.39 (0.74 ~ 2.59)			
minALC[2]					
G3-4		1.00			
G1-2	0.941	0.97 (0.45 ~ 2.08)			
preALC					
<1.1×10^9/L		1.00			
≥1.1×10^9/L	0.892	0.94 (0.36 ~ 2.46)			
PTV					1.048
≥210cm^3		1.00		1.00	
＜210cm^3	0.005	2.62 (1.35 ~ 5.10)	0.051	2.10 (1.00 ~ 4.41)	
EDIC					
<8.9Gy		1.00			
≥8.9Gy	<.001	0.31 (0.16 ~ 0.59)			
Lung V_5[3]					
＜46.5%		1.00			
≥46.5%	<.001	0.30 (0.15 ~ 0.60)			
Heart V_{10}					
＜60.5%		1.00			
≥60.5%	<.001	0.25 (0.13 ~ 0.48)			
SVC V_5					
＜96.0%		1.00			
≥96.0%	0.442	0.76 (0.37 ~ 1.54)			
Thoracic spine V_5					1.055
≥57.3%		1.00			

＜57.3%	<.001	6.22 (3.12 ~ 12.39)	<.001	5.55 (2.71 ~ 11.36)
Ribs $V_{0.5}$				
＜67.8%		1.00		
≥67.8%	<.001	0.29 (0.15 ~ 0.57)		
Sternum V_1				
＜99.9%		1.00		
≥99.9%	0.183	0.53 (0.21 ~ 1.35)		
Bone marrow $V_{0.5}$				
＜79.9%				
≥79.9%	<.001	0.23 (0.12 ~ 0.44)		

Acronyms: BMI, Body Mass Index; ECOG, Eastern Cooperative Oncology Group; ALC, Absolute lymphocyte count; PTV, Planning Target Volume; EDIC, Effective dose to immune cells; SVC, superior vena cava

[1] According to AJCC 8th. [2] The lowest ALC during RT (minALC) was identified as minALC and graded according to the Common Terminology Criteria for Adverse Events version 5.0. [3] V_x denotes the relative volume of specific organs receiving X Gy.
